# Total choline intake and working memory performance in adults with phenylketonuria

**DOI:** 10.1186/s13023-023-02842-y

**Published:** 2023-07-29

**Authors:** Meriah S. Schoen, Kelly M. Boland, Shawn E. Christ, Xiangqin Cui, Usha Ramakrishnan, Thomas R. Ziegler, Jessica A. Alvarez, Rani H. Singh

**Affiliations:** 1 Department of Human Genetics, Emory University School of Medicine, 101 Woodruff Circle, Suite 7130, Atlanta, GA 30322 USA; 2 Department of Psychological Sciences, University of Missouri, Columbia, MO USA; 3 Department of Biostatistics and Bioinformatics, Rollins School of Public Health, Emory University, Atlanta, GA USA; 4 Hubert Department of Global Health, Rollins School of Public Health, Emory University, Atlanta, GA USA; 5 Department of Medicine, Emory University School of Medicine, Atlanta, GA USA

**Keywords:** Phenylketonuria, PKU, Choline, Working memory, Executive function, Diet

## Abstract

**Background:**

Despite early diagnosis and compliance with phenylalanine (Phe)-restricted diets, many individuals with phenylketonuria (PKU) still exhibit neurological changes and experience deficits in working memory and other executive functions. Suboptimal choline intake may contribute to these impairments, but this relationship has not been previously investigated in PKU. The objective of this study was to determine if choline intake is correlated with working memory performance, and if this relationship is modified by diagnosis and metabolic control.

**Methods:**

This was a cross-sectional study that included 40 adults with PKU and 40 demographically matched healthy adults. Web-based neurocognitive tests were used to assess working memory performance and 3-day dietary records were collected to evaluate nutrient intake. Recent and historical blood Phe concentrations were collected as measures of metabolic control.

**Results:**

Working memory performance was 0.32 z-scores (95% CI 0.06, 0.58) lower, on average, in participants with PKU compared to participants without PKU, and this difference was not modified by total choline intake (F[1,75] = 0.85, *p* = 0.36). However, in a subgroup with complete historical blood Phe data, increased total choline intake was related to improved working memory outcomes among participants with well controlled PKU (Phe = 360 µmol/L) after adjusting for intellectual ability and mid-childhood Phe concentrations (average change in working memory per 100 mg change in choline = 0.11; 95% CI 0.02, 0.20; *p* = 0.02). There also was a trend, albeit nonsignificant (*p* = 0.10), for this association to be attenuated with increased Phe concentrations.

**Conclusions:**

Clinical monitoring of choline intake is essential for all individuals with PKU but may have important implications for working memory functioning among patients with good metabolic control. Results from this study should be confirmed in a larger controlled trial in people living with PKU.

## Background

Phenylketonuria (PKU; OMIM #261600) is a rare genetic metabolic disorder caused by mutations in the *PAH* gene and characterized by impaired phenylalanine (Phe) metabolism. Disruption of this pathway results in elevated blood Phe and reduced tyrosine (Tyr) concentrations, which lead to profound chemical and morphological changes in the brain [1]. These changes include reduced neurotransmitter synthesis [2], abnormalities in both white and gray matter [3, 4], and disruptions in the functional connectivity between brain regions [5, 6]. While these alterations manifest as severe developmental delays and intellectual disability among individuals with untreated PKU [7], early diagnosis and prompt initiation of a Phe-restricted diet has led to a remarkable improvement in prognosis [1]. Unfortunately, dietary treatment does not ameliorate all manifestations of the disorder. Many individuals with PKU still exhibit a slight decrease in overall intellectual functioning [8], in addition to circumscribed impairments in select cognitive domains including executive function [9–12]. Executive function refers to a collection of higher-order cognitive abilities such as problem solving, inhibitory control, task switching, and working memory [10].

Periods of poor metabolic control due to medical food (i.e., protein substitute) nonadherence and/or the consumption of excess intact protein likely contribute to these deficits. Previous research, however, suggests that current and historical fluctuations in Phe may account for only 43% of the variance in overall cognitive performance of patients with early-treated PKU [13]. This suggests that there are other relevant factors that may contribute to phenotypic variability. Beyond blood Phe levels, genotype, age, and inherent individual differences, these factors remain largely unknown [14].

To begin identifying additional drivers of cognitive variability, the present study investigated choline, which affects the brain through several pathways. Choline plays a critical role in cell membrane integrity (as a precursor for phospholipids) [15, 16], one-carbon metabolism (as a precursor for the methyl-donor betaine) [17], and neurotransmitter synthesis (as a precursor for acetylcholine) [18]. There also is evidence of cross-talk between choline and energy metabolism [19], which may subsequently affect neurocognition by modulating fat deposition and insulin sensitivity [20]. The importance of these functions for neurological development and sustained cognitive performance has been demonstrated in both animal and human studies, which have shown that higher prenatal choline intake has long-term benefits for attention and memory [21]. In children, this effect has been reported through seven years of age [22, 23], while rodent models have demonstrated improvement across the lifespan [24]. Evidence is more limited among healthy human adults, but one prior study found a positive association between concurrent choline intake and memory, in addition to an inverse association between prior choline intake and white matter hyperintensity volume [25]. Despite choline’s potential link to many of the pathophysiological changes in PKU (eg, demyelination, oxidative damage, differential methylation, impaired neural connectivity) [16, 26–28], few studies have investigated the relationship between choline and neurocognition in this population. The only prior research was conducted in a PKU mouse model, which evaluated choline in the context of a multinutrient supplement that also contained uridine monophosphate, docosahexaenoic acid, eicosapentaenoic acid, phospholipids, folic acid, selenium, and vitamins B12, C, B6, and E [29, 30]. Long-term use of this supplement was associated with improved hippocampal synaptic functioning and performance on a novel recognition task among mice maintaining low- or high-Phe diets [29, 30]. While there are presently no studies in human participants with PKU to complement these findings, our prior work using untargeted metabolomics identified substantial shifts in choline-related pathways among individuals with PKU relative to healthy controls [31]. Additionally, we have demonstrated that choline intake is suboptimal among both adults and children with PKU [32]. Even with the consumption of choline-fortified medical foods, only 10.8% of our previous PKU sample was able to achieve the sex- and age-specific adequate intake (AI) recommendations [33], which may increase susceptibility to neurocognitive deficits, and particularly memory impairment.

Given the importance of choline for cognitive function, the objective of this study was to evaluate the relationship between total choline intake and working memory performance in individuals with PKU. This study had two specific aims: (1) to determine if higher choline intake normalizes working memory performance in adults with PKU relative to healthy adults without PKU, and (2) to assess whether the relationship between total choline intake and working memory is dependent on metabolic control in participants with PKU.

## Materials and methods

### Participants

Adults 18–40 years of age with PKU (n = 42) were recruited primarily from the Genetics Clinic at Emory University and a database of research volunteers maintained by the Clinical Neuropsychology Lab at the University of Missouri. Recruitment flyers were additionally shared with patient advocacy organizations and registered dietitians at other metabolic clinics throughout the United States. Individuals were eligible if a PKU diagnosis was made and treatment was initiated shortly after birth, as substantiated by patient report or medical records. The PKU cohort was compared to a demographically-matched group of healthy adults without PKU (n = 41) based on age, sex, and years of education. These controls were recruited from the aforementioned Clinical Neuropsychology Lab database and the unaffected contacts of PKU participants. Participants with a history of neurologic compromise and major medical conditions unrelated to PKU (eg, closed head injury, multiple sclerosis) were excluded. Participants with PKU also were excluded if they were being treated with the enzyme substitution therapy pegvaliase (Palynziq®, BioMarin Pharmaceutical Inc, Novato, CA, USA). Out of the 83 enrolled participants, three (2 PKU, 1 non-PKU) did not complete the study due to scheduling conflicts. The final analytic sample comprised 40 participants with PKU and 40 healthy adults.

### Design and procedure

This was a cross-sectional study that was approved by the Research and Ethics Review Boards at Emory University and the University of Missouri, and informed consent was obtained from all participants. Participant visits were conducted over a HIPAA compliant cloud-based video and phone conferencing system. During each study visit, participants were asked to find a quiet, distraction-free location to complete a structured interview, cognitive tests, and diet record review. Participants were asked to report any distractions during the study visit that may have affected their performance.

The neurocognitive methods and associated data come from an initial study of working memory and anxiety in adults with early-treated PKU, which are detailed by Boland et al. [34]. To assess overall intellectual functioning, the Matrix Reasoning subtest from the Wechsler Adult Intelligence Scale 4th edition (WAIS-IV) [35] was administered. Visuospatial working memory and related executive functioning skills were assessed using four subtests from the web-based Cambridge Neuropsychological Test Automated Battery (CANTAB): Spatial Span (SSP), Paired Associates Learning (PAL), Rapid Visual Information Processing (RVP), and Spatial Working Memory (SWM) [36]. To evaluate verbal working memory, the Digit Span (DS) subtest from WAIS-IV also was administered. Each CANTAB subtest yielded age-, sex-, and education level-normed standard z-scores (*M* = 0, *SD* = 1). The overall score for the DS test was based on performance across the forward, backward, and sequencing trials. This score was then converted to an age-normed scaled score (*M* = 10, *SD* = 3), and subsequently transformed into a z-score to facilitate comparison with the other working memory tasks.

### Generation of a working memory composite

The z-scores from the four CANTAB visuospatial working memory tests (SSP, PAL, RVP, SWM) and the DS verbal working memory test were averaged to generate a composite working memory score. In cases where a participant’s score on an individual subtest was an outlier (> 2.5 SD relative to the sample mean) or a participant reported distractions in the testing environment that may have affected performance, the composite score was based on the remaining four subtests. This resulted in a single subtest being discarded for 6 participants (5 PKU, 1 non-PKU; no participants had more than one problematic subtest).

### Assessment of total choline intakes

In the three days prior to each study visit, PKU and non-PKU participants completed diet records with detailed descriptions of all foods, beverages, and supplements consumed. For participants with PKU, medical food consumption and prescriptions were also recorded. During each study visit, diet records were reviewed for accuracy and completeness by a trained research registered dietitian. To estimate the dietary and supplementary intake of choline and the other methyl-donor/co-factor nutrients that impact choline metabolism (vitamin B12, vitamin B6, folate, methionine, cysteine), dietary analysis was completed using the Nutrition Data System for Research (NDSR 2020*, *Nutrition Coordinating Center, University of Minnesota, Minneapolis, MN, USA). Medical foods were added to the NDSR database using manufacturer-supplied nutrient information, and low-protein modified foods were created using ingredients within the NDSR database that contained composition data for choline. Any additional items that were not found within the NDSR database were substituted for nutritionally comparable foods using the following set of nutrient tolerances per 100 g of food adapted from NDSR: 85 kcal, 2.5 g of fat, 100 mg of sodium, 10 g of carbohydrates, 1 g of protein, and 50 mg of Phe [37]. Total nutrient intakes were calculated by summing intakes from food, dietary supplements, and medical food (for the PKU sample). The contributions of food, medical food, and dietary supplements to total choline intake were calculated at the individual level by dividing a participant’s intake for each category by total choline intake.

Nutrient adequacy for choline, vitamin B12, vitamin B6, folate, methionine, and cysteine was determined using the cut-point method, which compares average nutrient intakes to the age- and sex-specific estimated average requirements (EAR) or adequate intake (AI) [38].

### Metabolic control measures

Multiple measures were used to assess metabolic control. To determine current Phe and Tyr concentrations, samples of capillary whole blood were collected via filter paper from participants on the day of cognitive assessment and sent to PerkinElmer Laboratories for analysis. For two participants who did not successfully submit filter papers, the most recent Phe measures (collected 1 and 17 days prior to the study visit) were used instead. Medical records also were obtained to gather historical Phe data. The highest available Phe concentration was then used to determine PKU biochemical phenotypes. Based on criteria adapted from Camp et al. [39] and Vockley et al. [40], three categories were defined: classical PKU (Phe > 1200 µmol/L), moderate PKU (Phe 600–1200 µmol/L), and mild PKU (Phe < 600 µmol/L). Mean Phe level for the year preceding study participation was calculated. As described previously by Christ et al. [41] and Brown et al. [42], an index of dietary control (IDC) was computed as the mean of all half-year median Phe concentrations for early childhood (0–5 years), middle childhood (6–11 years), adolescence (12–17 years), adulthood (18 + years), and across the lifetime (0-present). In the case of small gaps (eg, 1–2 years) in records, values were extrapolated using linear regression and Phe levels of adjacent years for purposes of IDC calculation. In cases of larger gaps, the participant was excluded from analysis of the relevant developmental epoch. Five of the 40 participants had significant gaps in historical Phe data and were excluded from the analysis for all developmental epochs.

### Statistical analysis

Sample characteristics and nutrient intakes were reported as median (interquartile range [IQR]) for continuous variables and frequency (percent) for categorical variables. Group profiles were compared using the Mann–Whitney U Test for continuous variables and the Chi-Square Test of Association for categorical variables. Analysis of Covariance (ANCOVA) was used to assess whether total choline intake modified working memory performance in PKU and non-PKU participants. The primary outcome was the working memory composite and the main predictors were diagnosis (PKU versus non-PKU) and total choline intake. For this analysis, total choline intake was converted into a binary variable based on previously published population averages [21]. The population average was utilized as an alternative AI, given the AI established by the Food and Nutrition Board of the National Academy of Medicine was based on a depletion-repletion study in adult men with liver damage as the endpoint for choline deficiency [33]. This is in contrast to the AIs for other nutrients, which are based on the estimated intake of a healthy group of individuals. Total choline intake greater than the age- and sex-specific population averages was considered high, and total choline intake below these averages was considered low. Demographic or dietary characteristics that were found to have a statistically significant difference between diagnosis groups, and which were associated with the working memory composite, were considered as covariates for the ANCOVA model. To be retained as a covariate, the variable was required to make a notable change in the magnitude (> 10%) or direction of the interaction (diagnosis*alternative choline AI) estimate. Only one variable, matrix reasoning (proxy for intellectual ability), met these criteria and was retained in the final model.

To evaluate the association between metabolic control and working memory performance, bivariate correlation analyses were conducted for concurrent Phe, Phe average from the year prior to the study, and all IDC measures (0–5 years, 6–11 years, 12–17 years, 18 + years, lifetime). To adjust for multiple comparisons, the false discovery rate (FDR) procedure of Benjamini and Hochberg was applied [43]. Multivariable linear regression was then used to evaluate if the relationship between total choline intake and working memory performance was dependent on metabolic control. For this analysis, both concurrent Phe and total choline intake were retained as continuous variables given data visualization identified linear trends, and this approach was preferable to prevent information loss in a reduced sample. Total choline intake was adjusted for total energy intake using the residual method [44], and the final model also was adjusted for overall intellectual ability (matrix reasoning) based on the aforementioned covariate criteria. To improve interpretation of the model, concurrent Phe concentrations were centered at 360 umol/L, while energy-adjusted choline intake and matrix reasoning scores were centered at sample means. Additionally, Phe and choline were rescaled to reflect a 50 umol/L difference in blood concentration and 100 mg difference in intake, respectively.

An additional multivariable regression analysis was conducted that adjusted for both current and historical metabolic control. IDC from middle childhood (6–11 years) was utilized as the historical measure of metabolic control given the present study identified a significant correlation between this IDC measure and the working memory composite. Given only 30 of the 40 participants had data for this IDC measure, and the correlation with working memory was not statistically significant after FDR adjustment, this was considered an exploratory analysis. Variance inflation factors were checked prior to conducting this analysis to ensure that excess collinearity between measures of metabolic control would not bias the regression estimates.

Given these regression analyses modeled choline differently than the ANCOVA in the full sample (continuous variable vs dichotomous variable, respectively), a sensitivity analysis was conducted to determine if both statistical approaches would produce consistent results. For this analysis, the aforementioned multivariable regression models (full sample and exploratory analyses) were evaluated with choline modeled as a dichotomous variable based on the alternative AI.

All statistical analyses were carried out in SAS 9.4 (version 9.4, SAS Institute, Cary, NC) and R (version 4.1.2). *P-*values (and q-values for the correlation analysis between Phe measures and working memory) ≤ 0.05 were considered statistically significant, and *p-*values > 0.05 and ≤ 0.10 were considered informative trends.

## Results

**Fig. 1:**
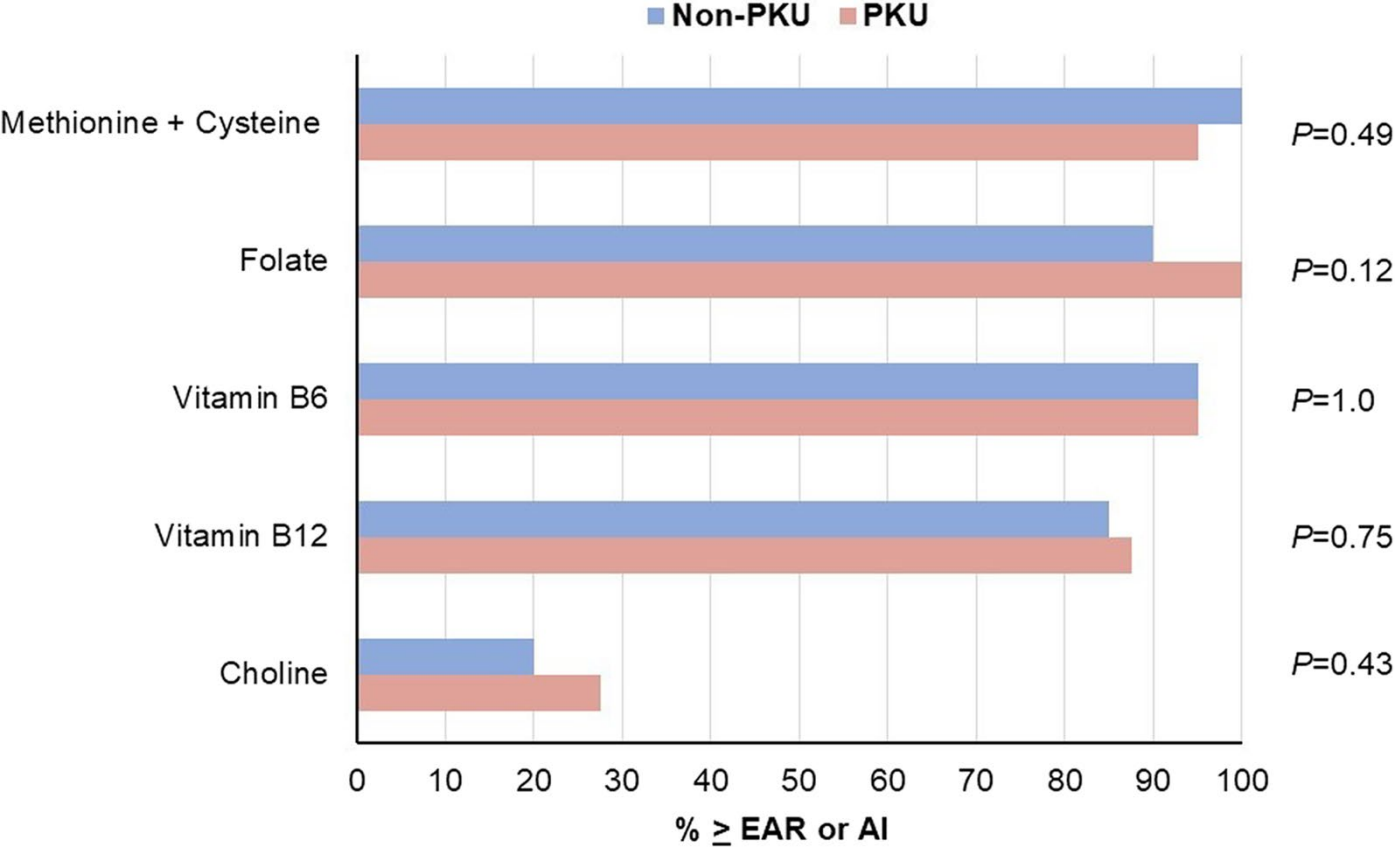
Percentages of the PKU and non-PKU cohorts with total intakes (food + medical food + dietary supplements) at or above the estimated average requirement (EAR) or adequate intake (AI) for choline and the other micronutrients/amino acids that affect choline metabolism (methionine + cysteine, folate, vitamin B12, vitamin B6). P-values reflect the difference in prevalence between the PKU and control groups as determined by the Chi-Square Test of Association or Fisher Exact Test

Characteristics of the PKU and non-PKU cohorts are reported in Table 1. The matched design resulted in similar demographic profiles, however, there were differences in dietary intake between the groups. Among the participants with PKU, median intakes of vitamin B6 and folate were notably higher, while intakes for total fat and the sum of methionine and cysteine were significantly lower than the non-PKU group. With regard to choline, there was a nonsignificant trend of lower median (IQR) total intake (PKU: 262.4 mg [188.7, 556.1], non-PKU: 315.8 mg [237.0, 418.4]; Mann Whitney U = 1555, *p* = 0.53) but higher choline adequacy (Fig. 1; PKU: 27.5%, non-PKU: 20%; *p* = 0.43). On average, fortified medical foods contributed 50.8% (SD: 32.4) of the total choline intake in participants with PKU, while 99.6% (SD: 2.7) of the choline intake in the non-PKU group was attributed to food sources. Dietary supplements were a poor source of choline for both groups. The average contribution was 0.07% (SD: 0.32) for participants with PKU and 0.45% (SD: 2.7) for participants without PKU.

**Table 1:** Sociodemographic and dietary profiles by diagnosis group^a^

	PKU (n = 40)	Non-PKU (n = 40)	*p*-value^b^
Female, n (%)	29.0 (72.5)	29.0 (72.5)	1.00
Age (years)	25.0 (21.0, 30.5)	26.0 (21.0, 30.5)	0.70
Education (years)	15.5 (14.0, 16.5)	16.0 (14.5, 17.0)	0.31
Intellectual ability (Matrix Reasoning Score)	11.0 (8.5, 13.0)	12.5 (10.5, 14.0)	0.03
BMI (kg/m^2^)	26.7 (23.0, 29.6)	23.7 (22.0, 27.1)	0.07
Total Energy (kcal/d)	1902.2 (1548.4, 2270.6)	1938.7 (1527.1, 2162.0)	0.78
Total Protein Intake (g/d)	74.1 (60.3, 89.5)	75.0 (61.5, 95.5)	0.39
Medical Food Protein (g/d)	48.9 (29.1, 66.0)	–	–
Intact Protein (g/d)	22.4 (15.9, 34.6)	–	–
Total Fat (g/d)	60.7 (50.6, 82.3)	78.0 (58.2, 91.2)	0.04
Total Choline (mg/d)	262.4 (188.7, 556.1)	315.8 (237.0, 418.4)	0.53
Total Vitamin B12 (mcg/d)	6.1 (3.3, 11.4)	5.5 (2.8, 8.4)	0.35
Total Vitamin B6 (mg/d)	3.3 (2.0, 4.0)	2.1 (1.5, 3.4)	0.02
Total Folate (mcg DFE/d)	1166.8 (815.9, 1479.1)	547.7 (394.1, 738.0)	< 0.0001
Total Methionine + Cysteine (mg/d)	2169.3 (1643.2, 2925.5)	2725.3 (2170.0, 3525.2)	0.01
Most Recent Phe (µmol/L)	362.4 (254.5, 585.9)	–	–
Sapropterin Dihydrochloride Treatment, n (%)	22.0 (27.5)	–	–
PKU Severity, n (%)^c^		–	–
Classical	4 (10.0)	–	–
Moderate	20 (50.0)	–	–
Mild	16 (40.0)	–	–

^a^Continuous variables are reported as median (IQR) and categorical variables are reported as frequency (%)

^b^*p*-values were derived from the Mann–Whitney U test for continuous variables and the Chi-Square Test of Association for categorical variables

^c^PKU severity defined as classical PKU (Phe > 1200 µmol/L), moderate PKU (Phe 600–1200 µmol/L), and mild PKU (Phe < 600 µmol/L)

Within the PKU cohort, 11 participants (27.5%) were managed with sapropterin dihydrochloride, a synthetic tetrahydrobiopterin treatment that can increase protein tolerance. However, only three of the 11 did not have medical food prescriptions. Out of the remaining 37 participants with medical food prescriptions, 90% used products that contained choline and other micronutrients and 72.5% were consuming the amount prescribed. Although 82.8% of the participants with PKU were consuming excess intact protein, median concentration for concurrent or most recent Phe was 362.4 µmol/L (IQR: 254.5, 585.9), which is only slightly above the therapeutic range (120–360 µmol/L).

### Comparison of working memory performance between participants with and without PKU

Relative to the non-PKU group, participants with PKU demonstrated a trend toward poorer performance on all working memory tests (Table 2); however, RVP was the only task that had a notable z-score difference (∆ = 0.37; 95% CI 0.10, 0.65) between study groups (F[1,77] = 7.21, *p* = 0.01, η_p_^2^ = 0.09). This trend was reflected in overall working memory performance, which was 0.32 z-scores (95% CI 0.06, 0.58) lower in participants with PKU compared to individuals without PKU (F[1,77] = 6.13, *p* = 0.02, η_p_^2^ = 0.07).

**Table 2:** Working memory performance in PKU and non-PKU participants^a^

Working memory outcome	Marginal mean Z-score (95% CI)			
	PKU (n = 40)	Non-PKU (n = 40)	Average Z-score difference (95% CI)	*p*-value
Composite	0.21 (0.04, 0.39)	0.53 (0.36, 0.71)	0.32 (0.06, 0.58)	0.02
SSP	0.10 (− 0.21, 0.41)	0.49 (0.18, 0.80)	0.39 (− 0.05, 0.83)	0.08
PAL	0.37 (0.05, 0.69)	0.80 (0.49, 1.12)	0.43 (− 0.02, 0.89)	0.06
RVP	− 0.01 (− 0.20, 0.18)	0.36 (0.17, 0.55)	0.37 (0.10, 0.65)	0.01
SWM	0.35 (− 0.01, 0.71)	0.59 (0.23, 0.95)	0.24 (− 0.27, 0.75)	0.36
DS	0.16 (− 0.12, 0.44)	0.41 (0.13, 0.70)	0.25 (− 0.15, 0.66)	0.22

DS, Digit Span; PAL, Paired Associates Learning; RVP, Rapid Visual Information Processing; SSP, spatial span; SWM, Spatial Working Memory

^a^Data Derived from ANCOVA adjusted for overall intellectual ability (Matrix Reasoning)

### Relationship between working memory performance and metabolic control

Correlations between measures of metabolic control and overall working memory are reported in Table 3. Concurrent/most recent Phe concentration was not significantly related to overall working memory performance (*r* = − 0.15, *p* = 0.37); however, in a subgroup with historical Phe data, there were moderate inverse correlations with IDC in middle childhood (6–11 years; n = 30, *r* = − 0.41, *p* = 0.02) and lifetime IDC (n = 16, r = − 0.59, p = 0.02). These correlations were no longer statistically significant after adjusting for multiple comparisons.

**Table 3:** Association between metabolic control measures and working memory performance

Metabolic control measure	N	Correlation coefficient (*r*)^a^	*p*-value	FDR Q-value^b^
Concurrent Phe	40	− 0.14	0.38	0.49
Previous Year Mean Phe	40	− 0.13	0.42	0.49
IDC Early Childhood (0–5 yrs)	25	− 0.19	0.37	0.49
IDC Middle Childhood (6–11 yrs)	30	− 0.41	0.02	0.09
IDC Adolescence (12–17 yrs)	29	− 0.25	0.20	0.46
IDC adulthood (18 + yrs)	26	− 0.14	0.49	0.49
IDC Lifetime (0-present)	16	− 0.59	0.02	0.09

IDC, Index of Dietary Control; Phe, Phenylalanine; Yrs, years

^a^Reflects the Spearman correlation coefficient

^b^Adjusted based on the procedure of Benjamini and Hochberg^43^

### Relationship between total choline intake, diagnosis, and working memory performance

**Fig. 2:**
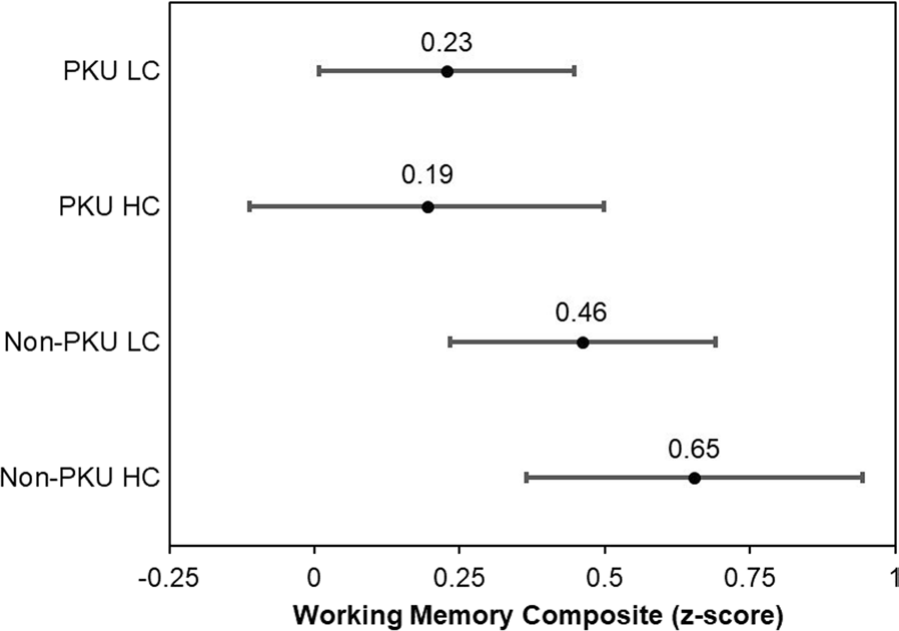
Interaction of choline consumption (low and high) and study group (non-PKU and PKU) based on ANCOVA for overall working memory performance (reflected by composite measure). Low and high choline consumption represent intake below and above the sex- and age-specific population averages, respectively. Estimated marginal means and 95% confidence intervals are reported for each group. This analysis did not identify choline consumption as a significant effect modifier (*p* = 0.36). Abbreviations: HC, high choline; LC, low choline

An ANCOVA was conducted in the full sample (PKU and non-PKU, N = 80) to assess whether choline intake above or below the sex- and age-specific population averages modified working memory performance in the PKU and non-PKU groups. After adjusting for intellectual ability (matrix reasoning), choline intake group was not a significant effect modifier or independently associated with working memory performance (Fig. 2; F[1,75] < 1, *p* > *0.05,* η_p_^2^ < 0.01 in both cases).

### Relationship between total choline intake, metabolic control, and working memory performance

Within the full PKU cohort (n = 40), multivariable regression was used to assess if the relationship between choline consumption and overall working memory performance was dependent on metabolic control (reflected by concurrent/most recent Phe concentrations). Given data visualization identified a linear trend between choline intake and working memory across different levels of metabolic control, choline was modeled on a continuous scale. After adjusting for intellectual ability, this analysis found that a 100 mg/day increase in energy-adjusted total choline intake was associated with an increase in working memory performance by 0.04 z-scores (95% CI − 0.05, 0.12; t[35] = 0.89, *p* = 0.38), on average, among individuals with well-controlled Phe concentrations (360 µmol/L). This association did not notably differ across the spectrum of Phe concentrations (t[35] = − 0.68, *p* = 0.50, Table 4). These findings were consistent with the results of our sensitivity analysis, which modeled total choline intake as a dichotomous variable (above or below sex- and age-specific population intake averages) and did not identify a main effect for choline intake group (β = 0.06 [95% CI − 0.34, 0.46], t[35] = − 0.3, *p* = 0.76) or significant effect modification by recent Phe (β = − 0.04 [95% CI − 0.11, 0.04], t[35] = − 1.02, *p* = 0.31).

**Table 4:** Results of the multivariable regression analyses of working memory performance with energy-adjusted total choline intake and metabolic control^a^

Model	N	Regression coefficient: choline intake (95% CI)^b^	*p*-value^c^	Regression coefficient: choline intake X Phe (95% CI)^d^	*p*-value^c^	Adjusted R^2^
1	40	0.04 (− 0.05, 0.1)	0.38	− 0.004 (− 0.02, 0.009)	0.50	0.36
2	30	0.10 (− 0.001, 0.19)	0.05	− 0.01 (− 0.02, 0.004)	0.16	0.44
3	30	0.11 (0.02, 0.20)	0.02	− 0.01 (− 0.02, 0.002)	0.10	0.52

^a^Adjusted for intellectual ability (matrix reasoning) in model 1 (full sample, n = 40) and model 2 (reduced sample, n = 30); adjusted for intellectual ability and IDC from 6 to 11 years in model 3 (exploratory analysis with reduced sample, n = 30). Matrix reasoning (MR), IDC, and energy-adjusted choline intake were centered at sample means (MR = 10, IDC = 353 µmol/L, choline = 366 mg) prior to inclusion in the regression models. Most recent Phe was centered at 360 µmol/L

^b^Regression coefficient represents the change in working memory performance per 100 mg/day increase in energy-adjusted total choline intake when Phe is optimal (360 µmol/L)

^c^Significance indicated at *p* ≤ 0.05; trends informative for future research indicated at *p* > 0.05 and ≤ 0.1

^d^Regression coefficient represents the amount by which the association between energy-adjusted total choline intake and working memory performance changes based on a 50 µmol/L increase in most recent Phe concentration

**Fig. 3:**
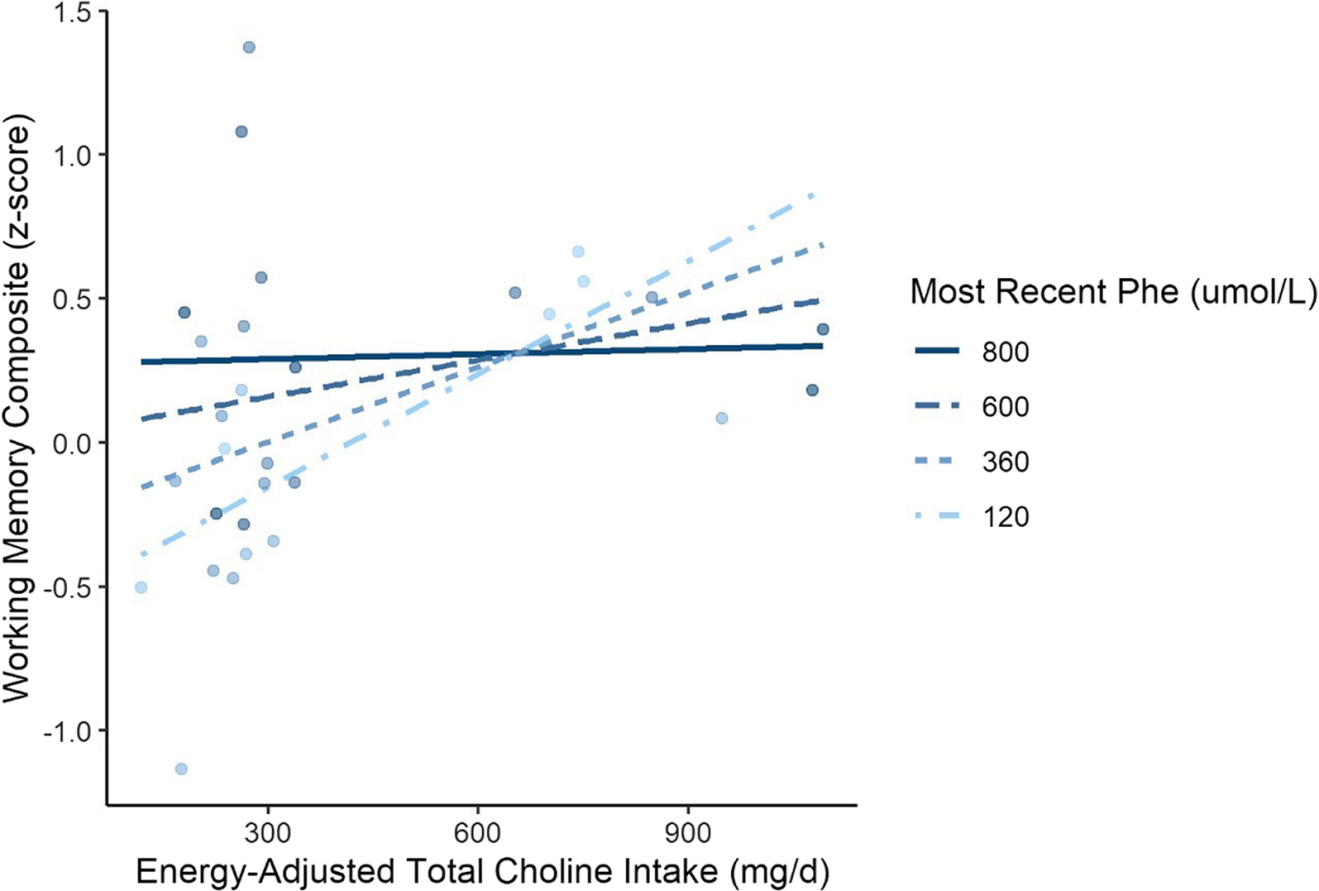
Association between working memory performance and energy-adjusted total choline intake across four levels of Phe concentration (120 µmol/L, 360 µmol/L, 600 µmol/L, 800 µmol/L) in a subsample of PKU participants with historical Phe data (n = 30). Data points represent observed data adjusted for intellectual ability (matrix reasoning) and IDC from 6 to 11 years

Given IDC from middle childhood (6–11 years) was correlated with working memory performance (Table 4), an exploratory multivariable regression analysis, which incorporated this measure as a covariate, was performed on a subgroup of 30 participants with available IDC data. After adjusting for intellectual ability and historical Phe, energy-adjusted choline intake was positively related to working memory performance. The average estimated improvement in working memory was 0.11 z-scores (95% CI 0.02, 0.20; t[24] = 2.41, *p* = 0.02) when total choline intake was increased by 100 mg/day. A relevant trend, which was not statistically significant, also became evident in the interaction between choline intake and metabolic control (β = − 0.01 [95% CI − 0.02, 0.002], t[24] = − 1.72; *p* = 0.10), which suggested that the benefit associated with choline consumption decreased as Phe concentrations increased (Table 4, Fig. 3). A similar, albeit non-significant, trend also was observed in our sensitivity analysis, which indicated that working memory performance was 0.36 z-scores (95% CI − 0.06, 0.78; t[24]:1.78; *p* = 0.09) higher among adults with well controlled PKU whose total choline intake was above the population mean. In alignment with the continuous parameter model, the difference between choline intake groups diminished as Phe concentrations increased (β = − 0.07 [95% CI − 0.15, 0.004], t[24]: − 1.94; *p* = 0.06).

To assess whether the identified effects in our exploratory regression analysis could be attributed to the reduced sample (n = 30 instead of n = 40 in original model) rather than the adjustment of historical Phe, we conducted an additional multivariable analysis on the subsample of 30 without including IDC as a covariate. This reduced model explained a greater proportion of the variance in working memory performance than the original model (Adjusted R^2^ Original Model = 0.36, Adjusted R^2^ Reduced Model = 0.44) and the main effect for choline reached statistical significance (β = 0.10 [95% CI − 0.001, 0.19], t[25] = 2.05, *p* = 0.05). To evaluate the sources of these changes, characteristics of the reduced sample and the 10 participants with missing IDC data were compared (Table 5). There were no characteristics that notably differed between the groups. Further inspection of the regression diagnostics identified one participant in the original model whose characteristics differed from the pattern identified in the majority of the sample (Cook’s D > 0.1). Despite having low Phe concentrations (261 µmol/L) and very high choline intake (777.9 mg/d), this participant had poor working memory performance (z-score = − 0.09). Removal of this participant due to unavailable IDC data may have impacted the reduced model, however, adjusting for historical Phe explained an additional 8% of the variance in working memory performance.

**Table 5:** Comparison of characteristics between participants with and without IDC data from 6 to 11 years of age^a^

	With IDC (n = 30)	Without IDC (n = 10)	*P*-value^b^
Female, n (%)	9 (30)	2 (20)	0.70
Age (years)	25 (21, 30)	25 (21, 33)	0.94
Education (years)	16.0 (14.0, 17.0)	15.0 (15.0, 16.0)	0.69
BMI (kg/m^2^)	27.1 (22.7, 29.7)	24.8 (23.2, 28.3)	0.86
Intellectual Ability (Matrix Reasoning Score)	11.0 (9.0, 13.0)	10.0 (7.0, 12.0)	0.43
Most Recent Phe (µmol/L)	384.0 (239.6, 597.7)	335.8 (296.4, 574.1)	0.94
Total Protein Intake (g/d)	74.9 (61.1, 88.4)	67.1 (54.7, 90.6)	0.66
Medical Food Protein (g/d)	22.4 (15.8, 35.8)	23.8 (16.7, 32.0)	0.38
Intact Protein (g/d)	50.0 (38.4, 66.0)	42.0 (18.0, 60.0)	0.75
Total Fat (g/d)	60.2 (50.7, 87.4)	62.6 (46.9, 76.5)	0.94
Total Choline (mg/d)	255.1 (180.6, 510.0)	308.0 (196.8, 671.2)	0.51
Total Vitamin B12 (mcg/d)	5.4 (2.8, 11.4)	7.8 (5.2, 12.7)	0.18
Total Vitamin B6 (mg/d)	3.1 (2.0, 11.3)	3.7 (2.6, 3.9)	0.63
Total Folate (mcg DFE/d)	1083.3 (704.7, 1353.9)	1290.3 (1080.4, 1597.2)	0.16
Total Methionine + Cysteine (mg/d)	2216.7 (1676.0, 2767.7)	2091.7 (1610.3, 3093.3)	0.79
Adherence to Medical Food RX^c^	1.1 (0.8, 1.0)	1.0 (1.0, 1.0)	0.96
Adherence to Intact Protein RX^d^	1.8 (1.1, 2.2)	1.6 (1.2, 3.3)	0.72
Sapropterin Dihydrochloride Treatment, n (%)	16 (53.3)	6 (60.0)	1.00

^a^Continuous variables are reported as median (IQR) and categorical variables are reported as frequency (%)

^b^*p*-values were derived from the Mann–Whitney U test for continuous variables and the Fisher Exact Test for categorical variables

^c^Calculated as the ratio of medical food protein equivalents consumed to medical food protein equivalents prescribed. Calculated for n = 28 for ‘With IDC’ group and n = 9 for ‘Without IDC’ group. Two participants with IDC and one participant without IDC did not have medical food prescriptions

^d^Calculated as the ratio of intact protein (g) consumed to intact protein (g) prescribed

## Discussion

Due to the many pathways by which choline impacts the structure and function of the brain [45], and the growing evidence to support choline’s long-term effects on memory performance [25, 46, 47], this study examined the relationship between total choline intake and working memory in adults with PKU and a demographically-matched group of individuals without PKU. Consistent with prior research [48, 49], participants with PKU demonstrated poorer overall working memory performance than non-PKU participants. However, overall working memory performance did not differ between participants with high versus low total choline intake, and this finding was not modified by diagnosis. This null finding may be attributed to the overall good nutriture among both PKU and non-PKU participants. When comparing intakes across the main micronutrients/amino acids that impact choline metabolism (vitamin B12, vitamin B6, folate, methionine, and cysteine), fewer than 15% of both groups were not meeting the EAR for all nutrients. Only 20% of controls and 27.5% of participants with PKU were meeting the AI for choline; however, this is higher than the prevalence reported by previous research in the US population (based on data from the National Health and Nutrition Examination Survey) [50, 51] and in our prior study in a larger sample of individuals with PKU [32]. In the present sample of participants with PKU, the improved nutrient density that we observed may derive from adherence to medical food prescriptions (found in 72.5% of sample) and the consumption of medical foods that contained vitamins and minerals (found in 90% of sample). As choline is concentrated in protein-rich foods [52], and most participants were consuming more intact protein than prescribed, this could also enhance overall nutrient adequacy. The similar pattern observed in the non-PKU group may be attributed to advanced education (bachelors or masters degrees), which was reported by 65% of the participants and has previously been associated with better health-related behaviors [53].

Underlying metabolic variation may also explain why our findings did not match our hypothesis. The majority of participants in our study were premenopausal women, and this group has a reduced dietary requirement for choline compared to men and postmenopausal women [54, 55]. This discrepancy can be attributed to the increased concentrations of estrogen in younger women, which regulate the expression of the *PEMT* gene. This gene encodes the phosphatidylethanolamine n-methyltransferase enzyme (PEMT) that is essential for the endogenous synthesis of choline [56], resulting in a reduced dietary requirement for choline. Beyond estrogen, there are several single nucleotide polymorphisms (SNPs) that have been found to modulate choline biosynthesis [57]. While this study did not assess genetic variation, it is possible that this sample did not include many individuals with these functional SNPs. Hence, it may not have been possible to identify an association between choline intake and working memory if this sample contained few individuals with both metabolic inefficiencies and low total choline intake [58].

Among participants with PKU, this study did not find a notable relationship between working memory performance and recent Phe concentrations. While this pattern aligns with the findings of a few prior studies [59, 60], there has not been a consistent relationship between working memory and metabolic control in the literature [61, 62]. This may reflect different methods of measuring and defining working memory performance across studies, and the variable sensitivity of specific neurocognitive tasks to metabolic control. Nevertheless,, we identified a relationship between total choline intake and working memory in an exploratory analysis of 30 participants with PKU that had measures of metabolic control from adulthood and middle childhood (6–11 years). In this sub-group, increased total choline intake was associated with higher scores on the working memory composite among participants with good current metabolic control (Phe = 360 µmol/L) after adjusting for historical metabolic control and intellectual ability. Although no prior studies have evaluated the cognitive correlates of choline in PKU, this finding complements the results of two previous studies in healthy adults. One was an observational study that found higher performance on verbal and visual memory tasks with increased concurrent choline intake [25]. The second was a randomized, double-blind, crossover trial that found improved processing speed, working memory, verbal learning, verbal memory, and executive function among participants who demonstrated poor baseline performance and were supplemented with 5’-diphosphocholine (CDP-choline; a derivative of choline used for phospholipid biosynthesis) [63].

This analysis also identified a statistically nonsignificant trend suggesting that that the positive association between choline intake and working memory is attenuated as Phe concentration increases. These findings contrast those of Bruinenberg et al. [30], who found that supplementation with a choline-containing multi-nutrient complex improved memory in PKU BTBR^Pah2^ mice with high Phe concentrations. We also evaluated the adequacy of other nutrients that are involved in choline metabolism (vitamin B12, B6, folate, methionine, cysteine), but did not identify any deficiencies or associations between these nutrients and working memory performance in the present sample. There are, however, several other nutrients that have been positively associated with working memory in adults (eg, cholesterol, alcohol, vitamin E, palmitoleic acid, oleic acid, alpha-linoleic acid, linoleic acid, vitamin C, vitamin D) [64], and many are provided in the medical foods and dietary supplements regularly consumed by individuals with PKU. Future research, which systematically examines the additive effect of choline with other neurologically relevant nutrients on working memory and associated cardiometabolic risk factors may improve the formulation and efficacy of these products.

It also is possible that we did not see any association between choline intake and working memory performance as Phe concentrations increased due to the potential impact of the phenylketone, phenylacetate, on PEMT. Phenylacetate concentrations are increased in patients with poor metabolic control [65], and one prior study found that that this metabolite has antiestrogenic properties, which could have implications for PEMT [66]. This may substantially increase the dietary requirement for choline among premenopausal women with PKU and poor metabolic control. As few individuals in this sample had very high choline intake in combination with high Phe, this hypothesis could not be further investigated.

While several prior studies have identified deficiencies for neurologically-relevant nutrients in PKU [67], few have evaluated how these nutrients affect cognitive outcomes in this population. This study is the first to evaluate the association between working memory and choline intake in individuals with PKU. A strength of this study was the use of a comprehensive approach to assessing working memory, including both visual and verbal components. This minimized the potential heterogeneity associated with using a single task. This study also used remote assessment for gathering cognitive outcomes, which expanded this study’s enrollment to clinics across the US without adding a travel burden for participants. By allowing for a more diverse sample, this methodology also increased the generalizability of our findings.

With these strengths, this study also had several limitations. With regard to the dietary data, we cannot eliminate the potential for self-reporting bias, which could have resulted in the over- or underestimation of our main variable of interest. This bias may have been eliminated with the use of a biochemical marker of choline status, which was not feasible for the present study. Another limitation was the incomplete IDC data. This was important given there was a significant unadjusted association between IDC from six to 11 years and working memory in the present study, and mid-childhood represents a key period during which working memory performance significantly changes [68]. For some patients, missing IDC data reflected nonadherence to the PKU monitoring guidelines. For others, the missing data reflected patient movement between clinics (with subsequent loss of records) or the switch to electronic charting, which enhanced the difficulty of accessing paper charts that contained early-life Phe concentrations.

## Conclusions

While the nutrient status of patients with poor metabolic control typically receives more attention, this study found that increased total choline intake was related to improved working memory outcomes among adults with well controlled PKU. Given this observation was based on a subgroup of participants with historical Phe data, future prospective controlled trials with larger samples and longitudinal data are needed to confirm the relationship between choline intake, Phe concentrations, and working memory performance. Nevertheless, this preliminary evidence suggests that there may be important cognitive benefits of closely monitoring choline intake among patients with Phe levels within therapeutic range.

## Data Availability

The dietary-related (choline intake) data may be available from the corresponding author (MSS), upon reasonable request. The neurocognitive data in the present study was collected as part of a larger study of working memory and psychoemotional functioning. The neurocognitive data may be available from the senior author (SEC) of that investigation, upon reasonable request. The data are not publicly available due to restrictions related to internal review board policies and informed consent limitations under which the data was originally collected.

## Abbreviations

AI: Adequate intake
CANTAB: Cambridge Neuropsychological Test Automated Battery
DS: Digit span
EAR: Estimated average requirement
IDC: Index of dietary control
PAL: Paired associates learning
PEMT: Phosphatidylethanolamine n-methyltransferase
Phe: Phenylalanine
PKU: Phenylketonuria
RVP: Rapid visual information processing
SD: Standard deviation
SNPs: Single nucleotide polymorphisms
SSP: Spatial span
SWM: Spatial working memory
Tyr: Tyrosine
WAIS-IV: Weschler Adult Intelligence Scale 4th edition
